# Multi-Ancestry Causal Association between Rheumatoid Arthritis and Interstitial Lung Disease: A Bidirectional Two-Sample Mendelian Randomization Study

**DOI:** 10.3390/jcm13206080

**Published:** 2024-10-12

**Authors:** Bo-Guen Kim, Sanghyuk Yoon, Sun Yeop Lee, Eun Gyo Kim, Jung Oh Kim, Jong Seung Kim, Hyun Lee

**Affiliations:** 1 Division of Pulmonary Medicine, Department of Internal Medicine, Kangbuk Samsung Hospital, Sungkyunkwan University School of Medicine, Seoul 03181, Republic of Korea; kbg1q2w3e@gmail.com; 2 Basgenbio Inc., Seoul 04167, Republic of Korea; whysh313@gmail.com (S.Y.); kimeg9160@gmail.com (E.G.K.); jokim8505@gmail.com (J.O.K.); 3 Department of Medical Informatics, Jeonbuk National University, Jeonju 54907, Republic of Korea; 4 Department of Otorhinolaryngology, Jeonbuk National University, Jeonju 54907, Republic of Korea; 5 Division of Pulmonary Medicine and Allergy, Department of Internal Medicine, Hanyang University College of Medicine, 222-1, Wangsimni-ro, Seongdong-gu, Seoul 04763, Republic of Korea

**Keywords:** rheumatoid arthritis, interstitial lung disease, Mendelian randomization, causal effect, bidirectional

## Abstract

**Abstract: Background**: Rheumatoid arthritis (RA) is associated with diverse extra-articular manifestations, including interstitial lung disease (ILD). No previous studies have examined the bidirectional relationship between RA and ILD using the Mendelian randomization (MR) analyses. Therefore, we aimed to investigate this subject using a two-sample bidirectional MR method. **Methods**: We performed bidirectional two-sample MR using summary statistics from genome-wide association studies (GWASs). The data are publicly available, de-identified, and from European (EUR) and East Asian (EAS) ancestries. **Results**: A total of 474,450 EUR participants and 351,653 EAS participants were included for either forward or reverse MR analysis. In our primary analysis, we found significant evidence of an increased risk of ILD associated with RA among individuals of EUR ancestry (OR_MR-cML_ = 1.08; 95% confidence interval [CI] = 1.03–1.14; *p* = 0.003) and EAS ancestry (OR_MR-cML_ = 1.37; 95% CI = 1.23–1.54; *p* < 0.001). Additionally, the reverse MR showed significant evidence of an increased risk of RA associated with ILD among those of EUR ancestry (OR_MR-cML_ = 1.12; 95% CI = 1.05–1.19; *p* < 0.001). However, only one instrumental variable was selected in the EAS ILD GWAS, and there was no increased risk of RA associated with ILD in those of EAS ancestry (OR_MR-cML_ = 1.02; 95% CI = 0.91–1.14; *p* = 0.740). **Conclusions**: Our findings indicate that RA and ILD have a bidirectional causal inference when using the MR analysis of GWAS datasets. The findings are only relevant for genetic predisposition; thus, further research is needed to determine the impact of non-genetic predispositions.

## 1. Background

Rheumatoid arthritis (RA) is the most common connective tissue disease, characterized by systemic inflammation that gradually harms joints, specifically the small joints in the wrists, hands, and feet, and frequently leads to significant disability [1]. Over 17 million people worldwide were affected by RA in 2020, and the prevalence of RA is projected to continue to increase through 2050 [2].

RA frequently involves diverse extra-articular manifestations. Among these, interstitial lung disease (ILD) is the gravest condition, leading to heightened morbidity and mortality rates [1,3,4]. Subjects with both RA and ILD have a decreased quality of life, experience constraints in functional capabilities, and show greater utilization of healthcare services [5,6].

Because of the importance of ILD in RA, numerous previous studies have evaluated the prevalence, incidence, and associated risk factors of ILD in subjects with RA [3,7,8,9]. Some of these studies suggested that ILD may precede RA [10,11,12,13,14], although each of these conditions may precede the other in a bidirectional causal association. However, uncertainty persists regarding the causal nature of the bidirectional association because potential confounders and reverse causal relationships can influence observational conclusions.

Mendelian randomization (MR) analyses have the advantage of suitability to assess causal relationships. MR circumvents the issues of reverse causation or confounders that may impact conventional observational epidemiology by employing genetic variants as instrumental variables (IVs) [15]. Previously, MR has been widely applied to explore associations between RA and various diseases, including lung cancer [16], obstructive lung disease [17], and depression [18]. However, limited information is available on the relationship between RA and ILD using MR analyses. Only one study has examined the relationship between RA and idiopathic pulmonary fibrosis (IPF) using MR analyses [19]. That study did not include ILDs other than usual interstitial pneumonia (UIP) and did not evaluate the bidirectional causality between RA and ILD.

Therefore, we aimed to investigate the possible bidirectional association between RA and ILD using a two-sample bidirectional MR method.

## 2. Methods

### 2.1. Study Design

**Figure 1:**
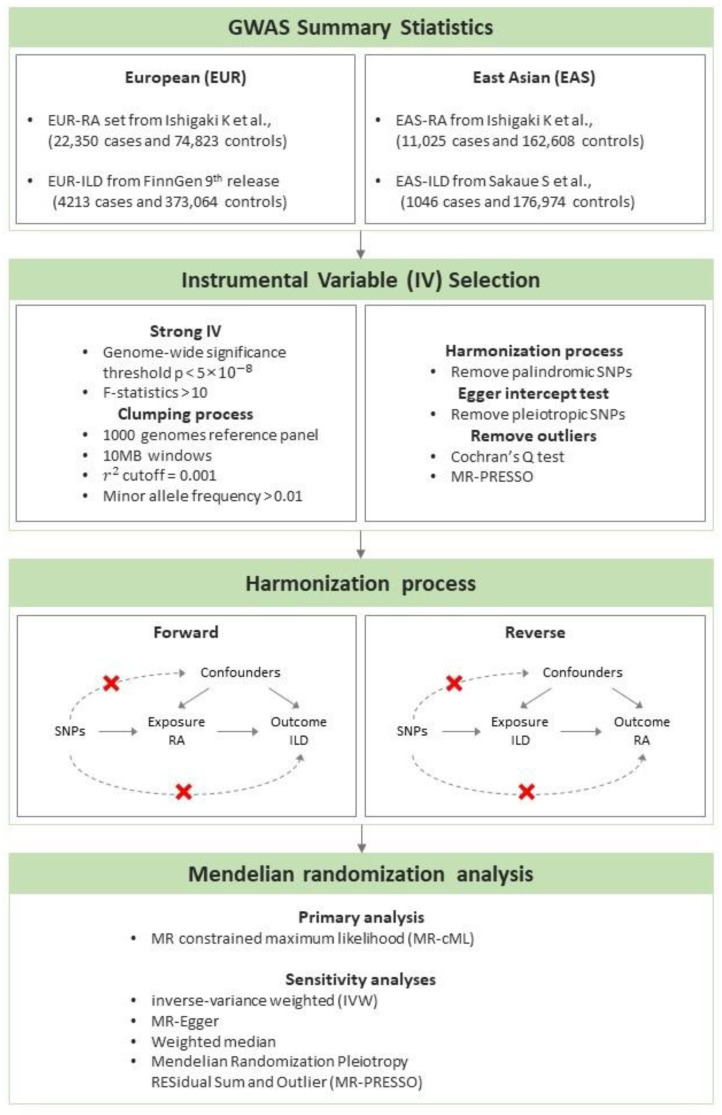
The workflow of the Mendelian randomization study to estimate the causal effect of rheumatoid arthritis on the development of interstitial lung disease in European and East Asian populations [23,24].

To investigate the relationship between RA and ILD in European (EUR) and East Asian (EAS) populations, we performed bidirectional two-sample MR using publicly available summary statistics of genome-wide association studies (GWASs) for EUR and EAS populations (Figure 1).

An MR study uses genetic variants as IVs to infer causality between the exposure and the outcome. Moreover, to reduce potential bias from confounding and reverse causation, genetic IVs must meet three core assumptions: (1) relevance—the genetic variant is associated with the risk factor; (2) independence—the genetic variant is not associated with confounders; and (3) exclusion restriction—the genetic variant influences the outcome only through the risk factor [20,21]. We performed MR analyses in accordance with the STROBE-MR guidelines [22].

This study was approved by the Institutional Review Board of Hanyang University Hospital (No. 2024-07-041) and performed in accordance with the principles of the Declaration of Helsinki. Written informed consent was waived because the GWAS database uses an anonymous patient identification system.

### 2.2. Data Sources

We used two different summary statistics from the GWAS datasets for each of the EUR and EAS ancestries. The summary statistics for RA were obtained from a multi-ancestry meta-analysis of GWASs by Ishigaki K et al. of eight EUR countries (Canada, the Netherlands, Sweden, the United States, the United Kingdom, France, Germany, and Spain) and three EAS countries, Biobank Japan (BBJ), China, and the Republic of Korea. The EUR-RA set included 22,350 RA cases and 74,823 controls; the EAS-RA set included 11,025 RA cases and 162,608 controls. All RA patients were diagnosed by professional rheumatologists [23].

The summary statistics for EUR-ILD were obtained from the FinnGen Consortium 9th release and included 4213 ILD cases and 373,064 controls (FinnGen id = finngen_R9_ILD_ENDPOINTS). For the EUR ILD GWAS, interstitial lung disease was defined as ICD-10 code J84, ICD-9 codes 515 and 516, and ICD-8 codes 48499 and 51701. For the EAS ILD GWAS, interstitial lung disease was defined as ICD-10 codes J84.1, J84.8, and J84.9. The summary statistics for EAS-ILD were obtained from the 220 deep-phenotype GWAS in BBJ by Sakaue S et al., which included 1046 ILD cases and 176,974 controls [24]. The BBJ project is an electronic medical record (EMR)-based prospective cohort that was launched in 2003 [25].

### 2.3. Instrumental Variables Selection

We selected the IVs based on the three core assumptions previously described. First, genetic variants associated with the exposure at the genome-wide significance threshold of *p* < 5 × 10^−8^ were selected. Then, genetic variants in linkage disequilibrium were pruned using an r^2^ of 0.001 within a 10 MB window, using the European 1000 genomes reference panel. Genetic variants were excluded if their minor allele frequency was less than 0.01. To avoid weak instrument bias, we calculated the F-statistics to assess the instrument’s strength. Genetic variants with F-statistics less than 10 were excluded from the analyses [26]. Pleiotropic genetic variants were detected and removed based on the MR-Egger intercept test. In the harmonization step, we removed palindromic variants with a minor allele frequency greater than the default threshold of 0.42. To improve the accuracy of the genetic IVs, we tested heterogeneity using Cochran’s Q test to detect outliers.

### 2.4. Statistical Analysis

MR analyses were performed in the forward and reverse directions in each of the EUR and EAS ancestries under the same analytic conditions. To increase our study’s robustness, we performed primary and sensitivity analyses based on different assumptions [27]. Estimates were presented as odds ratios (ORs) with 95% confidence intervals (CIs) and could be interpreted as the average change in the outcome per 2.72-fold increase in the prevalence of the respective binary exposure [28].

Primary analysis was performed with an MR-constrained maximum likelihood (MR-cML), a recently developed approach to account for both uncorrelated and correlated horizontal pleiotropy while effectively controlling for the type 1 error rate [29]. The MR-cML method is robust in the identification of violations of all three IV assumptions and outperforms the other MR methods.

The sensitivity analyses included the inverse-variance weighted (IVW), MR-Egger, weighted median, and Mendelian Randomization Pleiotropy RESidual Sum and Outlier (MR-PRESSO) methods. The IVW method led to the most efficient estimation under the assumption that all instrumental variables were valid. The MR-Egger method, a weighted linear regression of the gene–outcome coefficients, was conducted to adjust for horizontal pleiotropy. The weighted median method assumed that up to 50% of the genetic variants were invalid. The MR-PRESSO method, an extension of the IVW, removed variants based on their contributions to heterogeneity [20]. All statistical analyses were performed with R version 4.2.0 using “MendelianRandomization”, “TwoSampleMR”, and “MRPRESSO” packages. The main steps of our analysis are summarized in the Supplementary Methods.

## 3. Results

In total, 474,450 EUR participants and 351,653 EAS participants were included in either the forward or reverse MR analysis (Supplementary Table S1). In total, 55 IVs for EUR RA, 33 IVs for EAS RA, 10 IVs for EUR ILD, and 1 IV for EAS ILD were chosen, and all had F-statistics greater than 30 (Supplementary Table S2).

### 3.1. MR of RA on ILD

**Figure 2:**
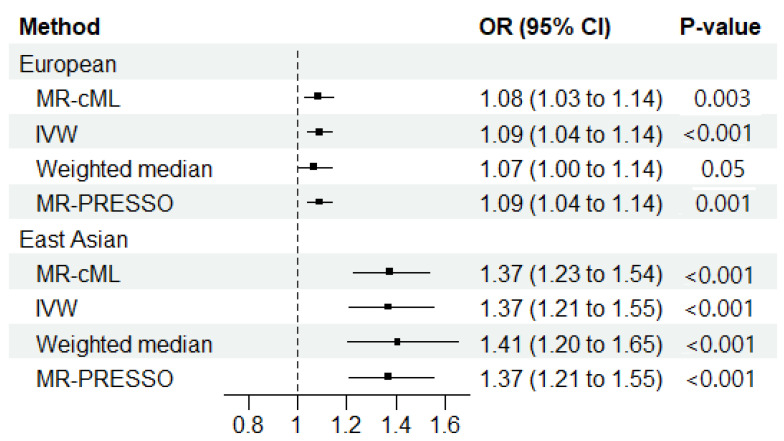
Mendelian randomization results regarding the causal effect of rheumatoid arthritis (RA) on the development of interstitial lung disease in European and East Asian populations. MR-cML, MR-constrained maximum likelihood; IVW, inverse-variance weighted; OR, odds ratio; 95% CI, 95% confidence interval; MR-PRESSO, MR pleiotropy residual sum and outlier.

We found a significantly increased risk of ILD associated with RA among individuals with EUR ancestry in our primary analysis (OR_MR-cML_ = 1.08; 95% CI = 1.03–1.14; *p* = 0.003) (Figure 2); additionally, no horizontal pleiotropy (Egger intercept: *p* > 0.05) or heterogeneity (Cochran’s Q: *p* > 0.05) was observed (Supplementary Tables S3 and S4). In the sensitivity analyses, an increased risk of ILD associated with RA was observed with the IVW (OR_IVW_ = 1.09; 95% CI = 1.04–1.14; *p* < 0.001) and the MR-PRESSO (OR_MR-PRESSO_ = 1.09; 95% CI = 1.04–1.14; *p* < 0.001) methods. However, only borderline significance was observed with the weighted median method (OR_Weighted median_ = 1.07; 95% CI = 0.99–1.14; *p* = 0.053) (Figure 2 and Supplementary Table S5).

In the primary analysis among the individuals of EAS ancestry, we observed an increased risk of ILD associated with RA (OR_MR-cML_ = 1.37; 95% CI = 1.23–1.54; *p* < 0.001) (Figure 2). No horizontal pleiotropy (Egger intercept *p* > 0.05) was observed, but significant heterogeneity was observed with Cochran’s Q test (Cochran’s Q: *p* < 0.011) (Supplementary Tables S3 and S4). However, in the sensitivity analyses of RA on ILD among those with EAS ancestry, significance was identified by the IVW (OR_IVW_ = 1.37; 95% CI = 1.21–1.56; *p* < 0.001), the weighted median (OR_Weighted median_ = 1.41; 95% CI = 1.20–1.65; *p* < 0.001), and the MR-PRESSO methods (OR_MR-PRESSO_ = 1.37; 95% CI = 1.21–1.56; *p* < 0.001) (Figure 2 and Supplementary Table S5).

### 3.2. MR of ILD on RA

**Figure 3:**
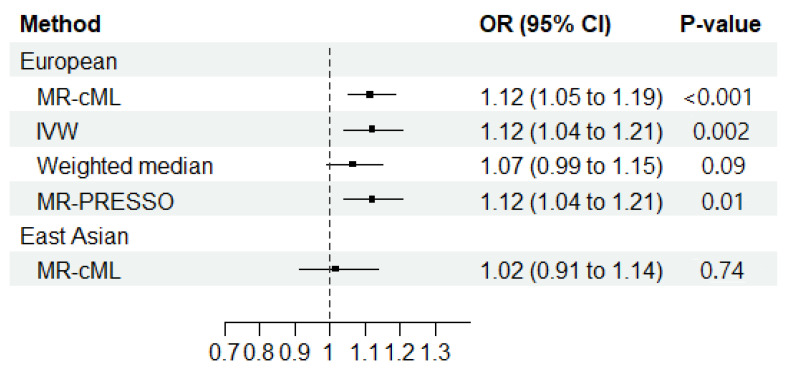
Mendelian randomization results regarding the causal effect of interstitial lung disease on the development of rheumatoid arthritis in European and East Asian populations. MR-cML, MR-constrained maximum likelihood; IVW, inverse-variance weighted; OR, odds ratio; 95% CI, 95% confidence interval; MR-PRESSO, MR pleiotropy residual sum and outlier.

The reverse-direction MR showed a significantly increased risk of RA associated with ILD among those with EUR ancestry (OR_MR-cML_ = 1.12; 95% CI = 1.05–1.19; *p* < 0.001) (Figure 3). No horizontal pleiotropy (Egger intercept: *p* > 0.05) or heterogeneity (Cochran’s Q: *p* > 0.05) was observed (Supplementary Tables S3 and S4). The sensitivity analyses of the reverse-direction MR among those with EUR ancestry showed an increased risk of RA associated with ILD with the IVW (OR_IVW_ = 1.12; 95% CI = 1.04–1.20; *p* = 0.002) and the MR-PRESSO methods (OR_MR-PRESSO_ = 1.12; 95% CI = 1.04–1.20; *p* < 0.014). However, no statistical significance was found with the weighted median method (OR_Weighted median_ = 1.07; 95% CI = 0.99–1.52; *p* = 0.092) (Figure 3 and Supplementary Table S5).

When using one instrumental variable for EAS ILD, no significant association was found between ILD and RA in the primary analysis (OR_MR-cML_ = 1.02; 95% CI = 0.91–1.14; *p* = 0.740) (Figure 3).

## 4. Discussion

In this study, we performed bidirectional two-sample MR to investigate the causal relationship between RA and ILD. To the best of our knowledge, this is the first demonstration of a causal relationship between RA and ILD in a bidirectional two-sample MR study. Our study revealed a bidirectional association between RA and ILD among individuals with EUR ancestry. Although we demonstrated an increased risk of ILD among those with EAS ancestry, we could not establish a reverse association, probably due to the small amount of data.

Evidence for an association between RA and ILD has been found in several studies. The prevalence of ILD ranges from approximately 4% to 8% in surveyed RA populations [3,7,8,9], and other studies have reported that the incidence of ILD varies from 1.8 to 7.1 per 1000 person-years [8]. Previous studies have shown that subjects with RA are more likely to develop ILD compared to those without. One USA population-based study reported that the risk of ILD was 7.7% for subjects with RA and 0.9% for non-RA subjects, a nearly nine-fold increase [3]. A combination of genetic predisposition and environmental factors might be involved in ILD in RA subjects. The most consistently reported risk factors include older age, male gender, smoking, the presence of positive anti-cyclic citrullinated peptide antibodies (anti-CCPs) or immunoglobulin M rheumatoid factor, and RA disease activity [30].

However, observational studies are limited in their ability to provide strong evidence of causality or the direction of causality, and they are susceptible to confounding and reverse causality. Because MR analysis is well suited to assess causal relationships, our study performed a two-way MR analysis using the GWAS dataset to provide information on the causal relationship between the two diseases in terms of genetic predisposition. From this view, the results of our study highlight the significant role of genetic predisposition in the development of ILD and RA. However, it is important to note that our findings do not provide evidence regarding responses to treatment, including various DMARDs and biologically active agents. This gap in the data suggests that treatment response may be influenced by genetic and epigenetic factors, which were not addressed in our analysis. Additionally, our analysis did not incorporate environmental factors, which could also play a crucial role in disease development and treatment response. Future research should aim to explore these aspects to better understand the interplay between genetic predisposition, environmental factors, and treatment efficacy. Incorporating such parameters could provide a more comprehensive understanding of disease mechanisms and improve personalized treatment strategies.

Some pathways could explain the development of ILD in RA. The immune response in RA occurs primarily in the synovial tissue of the joints. In some patients, citrullinated peptides are also produced in the lungs and can trigger an immune response, such as T- and B-cell infiltration into the lung interstitium [31]. Fibroblasts in the lungs are activated and differentiate into myofibroblasts under the influence of cytokines like IL-13 and tumor growth factor-β (TGF-β), with the resulting immune response causing lung fibrosis [31]. Another hypothesis is that aging alveolar epithelial cells gain the ability to secrete proteins, including growth factors (TGF-β), chemokines (CXCL12), and matrix metalloproteinases (MMP-7), that promote the formation of tissue fibrosis in patients with a genetic susceptibility to RA [31,32]. Consistent with our findings, this evidence suggests that RA may be a preceding factor in the development of ILD.

Contrary to the concept that RA precedes ILD, studies have suggested that ILD precedes RA by up to seven years [10,11,12,13,14]. For example, one matched cohort study of RA-with-ILD patients and RA-without-ILD patients found that approximately 14% of those with RA-ILD had been diagnosed with ILD one to five years before the diagnosis of RA [14]. Supporting this notion, another previous study analyzing the relationship between RA and IPF using MR showed a significant causal effect of IPF on seropositive RA [19]. This result suggests that some RA-UIP may be due to a cause–effect relationship between IPF and RA rather than a coincidental occurrence of IPF in subjects with RA. However, that study was limited in terms of evaluating RA-UIP only and did not account for correlated pleiotropy. Overcoming this limitation, we extended the previous findings by including RA-ILD patients other than those with RA-UIP (e.g., non-specific interstitial pneumonia, organizing pneumonia, and diffuse alveolar damage) [33] using MR-cML, a recently developed method. This new method is robust in identifying violations of both uncorrelated and correlated pleiotropy and has clarified the cause–effect relationship between ILD and RA [29].

Regarding the mechanism of ILD causation of RA, researchers suggested that citrullinated proteins in the lungs may act as antigenic targets for local immune cells, leading to anti-CCP formation and pulmonary interstitial involvement and triggering later RA development [34,35]. However, the exact mechanism by which RA may initiate in the lungs has not been elucidated. Further studies regarding the pathophysiology of the temporal development of ILD and articular involvement of RA are needed.

The major advantage of our study is that we confirmed the bidirectional association between RA and ILD using MR analyses. The epidemiological association regarding the bidirectional association between RA and ILD could not be confirmed since epidemiological studies cannot escape many potential biases that lead to reverse causalities. Accordingly, our study emphasized the importance of the surveillance of ILD and joint symptoms in subjects with RA. Regarding future clinical implications, the use of GWAS technology holds significant promise for providing individualized therapy for patients with RA. By identifying genetic markers associated with both RA and its extra-articular complications, clinicians can tailor treatments to minimize these risks. Personalized therapeutic strategies based on genetic predisposition could enhance treatment efficacy and improve patient outcomes by addressing the specific genetic factors contributing to disease progression and complications.

Despite these advantages, there were several limitations to our study. First, our analyses could not be extended to ancestries other than EUR and EAS due to limited data availability. Second, the RA and the ILD datasets used in our EAS analyses had a partial overlap; both included the BBJ. Third, as only one IV was available for the EAS MR effect of ILD on RA, sensitivity analyses requiring multiple SNPs could not be performed. Therefore, the MR analysis had low statistical power. Fourth, due to the relatively small sample size of individuals with RA having seropositive status among the ILD GWAS data, subgroup analysis based on RA seropositivity was not performed. When larger-scale GWAS data become available in the future, studies should consider incorporating seropositivity in the analyses. Fifth, as information regarding some clinically relevant factors (e.g., smoking status, RA treatment, etc.) was not available, we could not incorporate these factors into our analysis. Future studies evaluating this subject should consider including these relevant factors in the analyses.

In conclusion, our study suggested a bidirectional causal effect between RA and ILD based on an MR analysis using GWAS datasets. As our findings only present the results for genetic predisposition, the shared pathogenesis between the two diseases indicates the importance of further studies incorporating other factors that may impact the natural course of either disease.

## Data Availability

We used two different summary statistics of GWAS datasets for each of the European (EUR) and East Asian (EAS) ancestries. Summary statistics for rheumatoid arthritis were obtained from a multi-ancestry meta-analysis of GWASs by Ishigaki K et al. of eight EUR countries (Canada, the Netherlands, Sweden, the United States, the United Kingdom, France, Germany, and Spain) and three EAS countries, Biobank Japan (BBJ), China, and the Republic of Korea. The EUR-RA set included 22,350 RA cases and 74,823 controls; the EAS-RA set included 11,025 RA cases and 162,608 controls. All RA patients were diagnosed by professional rheumatologists. Summary statistics for EUR-interstitial lung disease (ILD) were obtained from the FinnGen Consortium 9th release and included 4213 ILD cases and 373,064 controls (FinnGen id = finngen_R9_ILD_ENDPOINTS). Summary statistics for EAS-ILD were obtained from the 220 deep-phenotype GWAS in BBJ by Sakaue S et al., which included 1046 ILD cases and 176,974 controls. The BBJ project is an electronic medical record (EMR)-based prospective cohort that was launched in 2003.

## Supplementary Materials

The following supporting information can be downloaded at: https://www.mdpi.com/article/10.3390/jcm13206080/s1, Table S1. Summary statistics generated in MR analysis; Table S2. Harmonized instrumental variables; Table S3. Horizontal pleiotropy of instrumental variables; Table S4. Heterogeneity test; Table S5. Results of causal estimation and sensitivity analyses.

## Abbreviations

anti-CCPs	anti-cyclic citrullinated peptide antibodies
BBJ	Biobank Japan
CIs	confidence intervals
EAS	East Asian
EUR	European
GWASs	genome-wide association studies
IPF	idiopathic pulmonary fibrosis
ILD	interstitial lung disease
IVs	instrumental variables
IVW	inverse-variance weighted
MR	Mendelian randomization
MR-cML	MR-constrained maximum likelihood
MR-PRESSO	Mendelian Randomization Pleiotropy RESidual Sum and Outlier
ORs	odds ratios
RA	rheumatoid arthritis
TGF-β	tumor growth factor-β
UIP	usual interstitial pneumonia
