# Supplementary material for: Multi-Ancestry Causal Association between Rheumatoid Arthritis and Interstitial Lung Disease: A Bidirectional Two-Sample Mendelian Randomization Study
Source: J Clin Med. 2024 Oct 12;13(20):6080. doi: 10.3390/jcm13206080 (PMC11508725; doi:10.3390/jcm13206080)
Supplement: Supplementary file 1 [file jcm-13-06080-s001.zip › jcm-3233297-supplementary.pdf]

**Supplementary Methods**

The analysis performed in this study involved Mendelian Randomization (MR) to explore the bidirectional association between rheumatoid arthritis (RA) and interstitial lung disease (ILD) using European (EUR) and East Asian (EAS) ancestry data. The key steps of the analysis are outlined below, and the corresponding R code is available upon request.

**S1. Software information**

The analysis was conducted using R version 4.2.0, utilizing the MendelianRandomization, TwoSampleMR, and MRPRESSO packages.

**S2. Data preprocessing**

The summary statistics for RA and ILD were imported from publicly available GWAS datasets; EUR RA url: https://www.ebi.ac.uk/gwas/studies/GCST90132223, EAS RA url: https://www.ebi.ac.uk/gwas/studies/GCST90132224, EUR ILD url: https://risteys.finregistry.fi/endpoints/ILD_ENDPOINTS, and EAS ILD url: https://pheweb.jp/pheno/ILD. The datasets were cleaned, and significant SNPs were filtered based on a genome-wide significance threshold ($p < 5e-8$).

```
read_ra_eur <- data.table::fread(dir_ra_eur)
read_ild_eur <- data.table::fread(dir_ild_eur)
ra_sig_eur <- read_ra_eur %>%
  filter(p_value < 5e-8)
ra_sig_eur$phenotype <- "Rheumatoid arthritis_eur"
```

```
ra_sig_eur <- format_data(
        dat = ra_sig_eur,
        type = 'exposure',
        phenotype_col = "phenotype",
        snp_col = "variant_id",
        beta_col = "beta",
        se_col = "standard_error",
        effect_allele_col = "effect_allele",
        other_allele_col = "other_allele",
        pval_col = "p_value"
      )
```

**S3. Clumping and harmonization**

The significant SNPs were clumped using linkage disequilibrium thresholds to remove correlated SNPs. Data for RA and ILD were harmonized to ensure alignment of effect alleles and consistent interpretation across datasets.

```
clumped_ra_eur <- clump_data(ra_sig_eur, clump_kb = 10000, clump_r2 =
0.001, clump_p1 = 1, clump_p2 = 1, pop = "EUR")

out_ild_eur <- tryCatch({
  format_data(
    dat = read_ild_eur,
    type = 'outcome',
    snps = clumped_ra_eur$SNP,
```

```
      snp_col = "rsids",
      beta_col = "beta",
      se_col = "sebeta",
      effect_allele_col = "alt",
      other_allele_col = "ref",
      pval_col = "pval",
      eaf_col = 'af_alt'
    )
}, error = function(e) {
    NULL
})
out_ild_eur$outcome <- "Interstitial Lung Disease"

harmo_eur <- harmonise_data(clumped_ra_eur, out_ild_eur)
harmo_eur <- harmo_eur[harmo_eur$mr_keep != "FALSE", ]
```

**S4. MR and sensitivity analyses**

Several MR methods were applied, including the inverse-variance weighted (IVW) approach, MR-Egger, and MR-PRESSO to test for pleiotropy and causal inference. The MR-constrained maximum likelihood (MR-cML) method was utilized for the primary analysis.

```
res_presso_eur <- mr_presso(BetaOutcome = "beta.outcome",
BetaExposure = "beta.exposure", SdOutcome = "se.outcome", SdExposure
= "se.exposure", OUTLIERtest = TRUE, DISTORTIONtest = TRUE, data =
harmo_eur, NbDistribution = 2000,   SignifThreshold = 0.05)

res_presso_main_eur <- res_presso_eur$`Main MR results`

exp(res_presso_main_eur$`Causal Estimate`[2])
exp(res_presso_main_eur$`Causal Estimate`[2] - 1.96 *
```

```
res_presso_main_eur$Sd[2])
exp(res_presso_main_eur$`Causal Estimate`[2] + 1.96 *
res_presso_main_eur$Sd[2])

#Remove outlier rs3184504
harmo_eur <- harmo_eur[harmo_eur$SNP != "rs3184504", ]

#F-statistics
harmo_eas$F <- (harmo_eas$beta.exposure^2) /
(harmo_eas$se.exposure^2)

#MR-cML
res_cml_eur <-mr_cML(mr_input(bx = harmo_eur$beta.exposure, bxse =
harmo_eur$se.exposure, by = harmo_eur$beta.outcome, byse =
harmo_eur$se.outcome), num_pert=200, MA = TRUE, DP = TRUE, n =
100000)

exp(res_cml_eur@Estimate)
exp(res_cml_eur@Estimate - 1.96 * res_cml_eur@StdError)
exp(res_cml_eur@Estimate + 1.96 * res_cml_eur@StdError)

res_hetero_eur <- mr_heterogeneity(harmo_eur)
res_pleio_eur <- mr_pleiotropy_test(harmo_eur)

res_eur <- mr(harmo_eur, method_list =
c('mr_ivw','mr_egger_regression','mr_weighted_median'))

forestplot_eur <- mr_forest_plot(res_single_eur)
```

**Supplementary Tables**

Excel Sheet 2: **Table S1.** Summary statistics generated in MR analysis.

Excel Sheet 3: **Table S2.** Harmonized instrumental variables

Excel Sheet 4: **Table S3.** Horizontal pleiotropy of instrumental variables

Excel Sheet 5: **Table S4**. Heterogeneity test

Excel Sheet 6: **Table S5.** Results of causal estimation and sensitivity analyses
